# A noninvasive test for human prion disease using hair roots and scalp

**DOI:** 10.1038/s41598-025-25310-y

**Published:** 2025-11-24

**Authors:** Thi-Thu-Trang Dong, Hiroyuki Honda, Akio Akagi, Yasushi Iwasaki, Hitaru Kishida, Tadashi Tsukamoto, Kensaku Kasuga, Hirotsugu Takashima, Tatsuhiro Terada, Kensuke Ikenaka, Takeshi Ikeuchi, Tsuyoshi Mori, Hideki Mochizuki, Kenjiro Ono, Yoshihisa Takiyama, Tsuyoshi Hamaguchi, Hiroyuki Murota, Nobuo Sanjo, Takeshi Fujimoto, Michio Kitayama, Koji Fujita, Motohiro Yukitake, Shinsuke Fujioka, Noriyuki Nishida, Yoshio Tsuboi, Tetsuyuki Kitamoto, Masaki Takao, Masahito Yamada, Hidehiro Mizusawa, Katsuya Satoh

**Affiliations:** 1 Department of Acute Diseases and Emergency - Senior Official Institute, 108 Military Central Hospital, Hanoi, Vietnam; 2 Department of Neurology, Neuropathology Center, National Hospital Organization, Omuta National Hospital, Omuta, Japan; 3 Department of Neuropathology, Institute for Medical Science of Aging, Aichi Medical University, Nagakute, Japan; 4 Research Committee of Prion Disease and Slow Virus Infection, Research on Policy Planning and Evaluation for Rare and Intractable Diseases, Tokyo, Japan; 5 Department of Neurology, Yokohama City University Medical Center, Yokohama, Japan; 6 National Center of Neurology and Psychiatry, Kodaira, Japan; 7 Japan Prion Disease Surveillance Committee (J-PrD-SC), Tokyo, Japan; 8 Department of Molecular Genetics, Brain Research Institute, Niigata University, Niigata, Japan; 9 Department of Neurology, National Epilepsy Center, NHO Shizuoka Institute of Epilepsy and Neurological Disorders (NEC), Shizuoka, Japan; 10 Department of Biofunctional Imaging, Preeminent Medical Photonics Education & Research Center, Hamamatsu University School of Medicine, Hamamatsu, Japan; 11 Department of Neurology, Osaka University Graduate School of Medicine, Suita, Japan; 12 Division of Microbiology, Department of Infectious Diseases, Faculty of Medicine, University of Miyazaki, Miyazaki, Japan; 13 Department of Neurology, Graduate School of Medical Sciences, Kanazawa University, Kanazawa, Japan; 14 Department of Neurology, Fuefuki Central Hospital, Fuefuki, Japan; 15 Department of Neurology, Graduate School of Medical Sciences, University of Yamanashi, Kofu, Japan; 16 Department of Neurology, Kanazawa Medical University, Uchinada, Japan; 17 Department of Dermatology, Nagasaki University Graduate School of Biomedical Sciences, Nagasaki, Japan; 18 Joint Research Department of Rare intractable Neurological Disease Therapeutics Development Institute of Biomedical Engineering , Institute of Science Tokyo, 1-5-45 Yushima Bunkyo-ku, Tokyo, 113-8510 Japan; 19 Department of Neurology, Sasebo City General Hospital, Sasebo, Japan; 20 Department of Internal Medicine Kawasaki Medical School General Medical Center, Okayama, Japan; 21 Department of Neurology, Tokushima University Graduate School of Biomedical Sciences, Tokushima, Japan; 22 Department of Neurology, Long-Term Observation Research, Juntendo University, Tokyo, Japan; 23 Department of Molecular Microbiology and Immunology, Nagasaki University Graduate School of Biomedical Sciences, Nagasaki, Japan; 24 Division of CJD Science and Technology, Department of Prion Protein Research, Tohoku University Graduate School of Medicine, Sendai, Japan; 25 Division of Neurology, Department of Internal Medicine, Kudanzaka Hospital, Tokyo, Japan; 26 Unit of Medical and Dental Sciences, Department of Health Sciences, Nagasaki University Graduate School of Biomedical Sciences, 1-7-1 Sakamoto, Nagasaki, 852-8520 Japan; 27 Leading Medical Research Core Unit, Department of Brain Research Unit, Nagasaki University Graduate School of Biomedical Sciences, Nagasaki, Japan; 28 Department of Diagnostic Innovation Science, Center for Development of Advanced Medicine for Dementia, Obu, Japan; 29 Department of Neurology, Kouhoukai Takagi Hospital, International University of Welfare, Okawa, Japan; 30 Department of Pharmaceutical Sciences, International University of Health and Welfare, Okawa, Japan

**Keywords:** Prion disease, RT-QuIC assay, Scalp, Biomarkers, Hair root, Molecular neuroscience, Diagnostic markers, Neurological disorders, Biomarkers, Medical research, Molecular medicine, Oncology

## Abstract

Invasive tests like cerebrospinal fluid (CSF) examination are highly effective for diagnosing human prion disease (HPD). Real-time quaking-induced conversion (RT-QuIC) CSF assay demonstrates 80–90% sensitivity for HPD diagnosis. To establish a minimally invasive diagnostic approach, we evaluated RT-QuIC testing on hair root and scalp samples. We collected scalp samples from 22 HPD and 5 non-HPD patients during pathological examinations and analyzed them using RT-QuIC assay and neuropathological methods. In our prospective study, hair root and CSF samples from 300 patients were tested using RT-QuIC assay along with other biomarkers, including 14-3-3 protein, total tau protein, RT-QuIC CSF assay, and MRI findings. All 22 HPD patients demonstrated positive prion seeding activity in scalp and hair root RT-QuIC assays. Neuropathological examination in one HPD patient revealed abnormal prion protein in scalp tissue. Among 177 HPD patients diagnosed by Japan Prion Surveillance Committee and 123 non-HPD patients, RT-QuIC assay of hair roots demonstrated sensitivity and specificity of 45.8% and 100%, respectively. Sensitivities of 14-3-3 protein, total tau protein, RT-QuIC CSF assay, and MRI findings were 83.1%, 86.4%, 74.6%, and 100%, respectively, with specificities of 65.0%, 65.0%, 100%, and 56.1%, respectively. RT-QuIC assays could be developed into novel diagnostic methods for neurodegenerative diseases.

**Supplementary Information:**

The online version contains supplementary material available at 10.1038/s41598-025-25310-y.

## Introduction

Prion diseases are critical neurodegenerative conditions that occur in humans and animals. They are also known as transmissible spongiform encephalopathies^1^. Human prion diseases (HPDs) include Kuru, Creutzfeldt–Jakob disease (CJD) and its variations, Gerstmann–Sträussler–Scheinker (GSS) disease, and fatal familial insomnia^1^. Sporadic-type HPDs include sporadic CJD (sCJD) and variably protease-sensitive prionopathy. sCJD comprises six phenotypes and commonly presents with progressive dementia and myoclonus^2^. Survival duration varies considerably among different sCJD subtypes, with less common subtypes such as MM2C or MV2K often demonstrating prolonged disease courses that frequently exceed one year, unlike the rapid progression typically observed in MM1 cases. In most cases, classical and typical sCJD usually results in death within 6 months. sCJD diagnosis relies on clinical characteristics and typical anomalies in supporting clinical tests. These tests include an electroencephalogram (EEG) exhibiting periodic sharp-wave complexes and a brain magnetic resonance imaging (MRI) scan showing a strong basal ganglia signal on diffusion-weighted imaging (DWI). The DWI-MRI hyperintensity pattern can differentiate sCJD from other rapidly progressing dementia with high sensitivity and specificity^3^.

In 2001, the protein misfolding cyclic amplification method was developed as a detection assay for PrP^Sc^ aggregation^4^. It was further improved to reproduce and amplify PrP^Sc^ in microtubes. The PrP^Sc^ amplification assay was developed in 2011. A blinded study on patients with CJD showed that the real-time quaking-induced conversion (RT-QuIC) assay achieved over 80% sensitivity and 100% specificity^5^, indicating the enhanced diagnostic capability of the RT-QuIC assay for suspected CJD.

Application of the RT-QuIC assay to cerebrospinal fluid (CSF) sample panels from sCJD patients and non-HPD patients revealed a specificity of 99–100% and sensitivity of 80–90% for identifying sCJD patients. Recent new diagnostic criteria of CJD were based on clinical, CSF (14-3-3 protein, total tau [t-tau] protein, and RT-QuIC assay), EEG, and DWI findings on MRI^6^.

Therefore, detecting prion seeding activity (PSA) in the RT-QuIC assay of CSF samples is a diagnostic criterion for HPD^6^. We developed the RT-QuIC assay for CSF samples with HPD utilizing recombinant human prion protein (hu-1st generation QuIC)^6^. In recent years, various RT-QuIC assays have been reported, including the 1st-generation QuIC assay, which utilizes recombinant hamster protein (ha-1st-generation QuIC assay). At present, the second-generation QuIC assay, which employs recombinant hamster protein at a higher temperature, is commonly used.

Detecting PSA using the RT-QuIC assay of other materials is a diagnostic criterion for HPD^6^.

The 1st and 2nd generation QuIC assays, which were designed to detect PSA, exhibit significant methodological differences. The substrate is recombinant human PrP (23-231) or recombinant Syrian hamster PrP (23-230) in the 1st generation assay, but changes to truncated recombinant Syrian hamster PrP (90-228) in the 2nd. The 2nd-generation QuIC assay differs from its predecessor in several other key aspects. For example, it uses a modified buffer containing SDS, allows testing of larger CSF samples and brain homogenate, employs higher incubation temperatures with longer shake-rest cycles, features less frequent fluorescence measurements, and has a specified 60 h run time. These modifications enhance the assay’s sensitivity and specificity to detect prion diseases across various sample types, potentially improving the diagnostic and research applications.

Several researchers have attempted to detect PSA in human tissue samples from the skin, peripheral nervous, digestive, and olfactory mucosa^7–9^. However, these samples are invasive and, therefore, cannot be performed frequently. Consequently, there is considerable interest in identifying tissues that could be obtained noninvasively and with sufficient frequency to confirm diagnosis and identify the early disease stages. To our knowledge, the scalp and hair follicles have not been the focus of any prior prospective investigations that used RT-QuIC technology.

This prospective study aimed to improve the sensitivity and utility of RT-QuIC technology for scalp and hair root diagnostic testing. Abnormal prion protein and PSAs were previously identified in skin samples obtained from patients with sCJD (Supplementary Table S1 online)^10–12^. The sensitivity of the skin RT-QuIC assay was 100%; however, all previous studies used retrospective designs (Supplementary Table S1 online).

We propose a novel method that is safer and less invasive for collecting scalp tissue and hair root samples. We evaluated the performance of our novel RT-QuIC analysis procedure on scalp and hair root samples obtained from patients with HPD and non-HPD controls.

## Results

### SD50 in scalp tissue as determined by a 1st-generation QuIC assay

Table 1 shows the results of the SD50 analysis of scalp samples from HPD patients using a first-generation RT-QuIC assay. We analyzed native brain tissue and scalp samples from nine cases (sCJD MM1) (Nos. 1–9). The average log SD50 in “native” brain samples from the nine cases was 9.96 ± 0.15, and the average log SD50 in the scalp samples was 7.38 ± 0.21 log SD_50_/g of tissue. Previous studies stated that all samples of sporadic CJD patients exhibited a similar level of seeding activity (10^9.17–10.25^/g)^5^, so the log SD50 in “native” brain samples of this was similar to that reported in previous studies^5^.

**Table 1:** Prion seeding activities in “native” scalp and brain tissue from 22 patients with human prion disease.

Patient number	Sex	Age of onset (years)	Mutation of *PRNP*	PrP^Sc^ Subtype according to Parchi’s classification	Log SD_50_/g Tissue in the “native” scalp (mean ± SD)	Log SD_50_/g Tissue in the “native” brain (mean ± SD)
1	Female	80	None	Type 1	7.75 ± 0.35	10.25 ± 0.35
2	Female	50	None	Type 1	7.5 ± 0.35	10.00 ± 0.00
3	Female	68	None	Type 1	7.75 ± 0.00	10.00 ± 0.35
4	Male	81	None	Type 1	7.13 ± 0.18	10.13 ± 0.18
5	Female	86	None	Type 1	7.75 ± 0.71	9.75 ± 0.00
6	Male	64	None	Type 1	6.75 ± 0.35	9.88 ± 0.18
7	Male	72	None	Type 1	7.88 ± 0.17	9.50 ± 0.00
8	Female	84	None	Type 1	7.38 ± 0.53	10.00 ± 0.35
9	Male	79	None	Type 1	6.50 ± 0.35	10.13 ± 0.18
10	Female	80	None	Type 1	7.88 ± 0.17	NA
11	Female	71	None	Type 1	7.75 ± 0.35	NA
12	Male	72	None	Type 1	7.00 ± 0.00	NA
13	Female	81	None	Type 1	8.00 ± 0.35	NA
14	Male	66	None	Type 1	8.00 ± 0.47	NA
15	Male	75	None	Type 1 + 2C	7.42 ± 0.12	NA
16	Female	66	None	Type 1 + 2C	8.42 ± 0.59	NA
17	Male	70	None	Type 2 T	7.63 ± 0.53	NA
18	Female	72	None	Type 2 T	7.88 ± 0.53	NA
19	Female	77	V180I	Type 2	6.38 ± 0.18	NA
20	Male	79	V180I	Type 2	6.88 ± 0.18	NA
21	Female	83	V180I	Type 2	6.33 ± 0.24	NA
22	Female	84	V180I	Type 2	8.00 ± 0.00	NA

*PRNP*: human *prion protein*, *PRNP* is the human gene encoding for the major prion protein PrP, NA: not available, type 1 + 2C: type 1 and cortical form of type 2, type 2 T: the thalamic form of type 2, V180I: prion protein gene codon 180 mutation (V180I), SD, standard deviation.

*All patients were homozygous for methionine (M) at codon 129 in the prion protein (*PRNP*) gene.

**This study was performed using a 1st-generation QuIC assay.

### Pathological findings of an MM1-patient

**Fig. 1:**
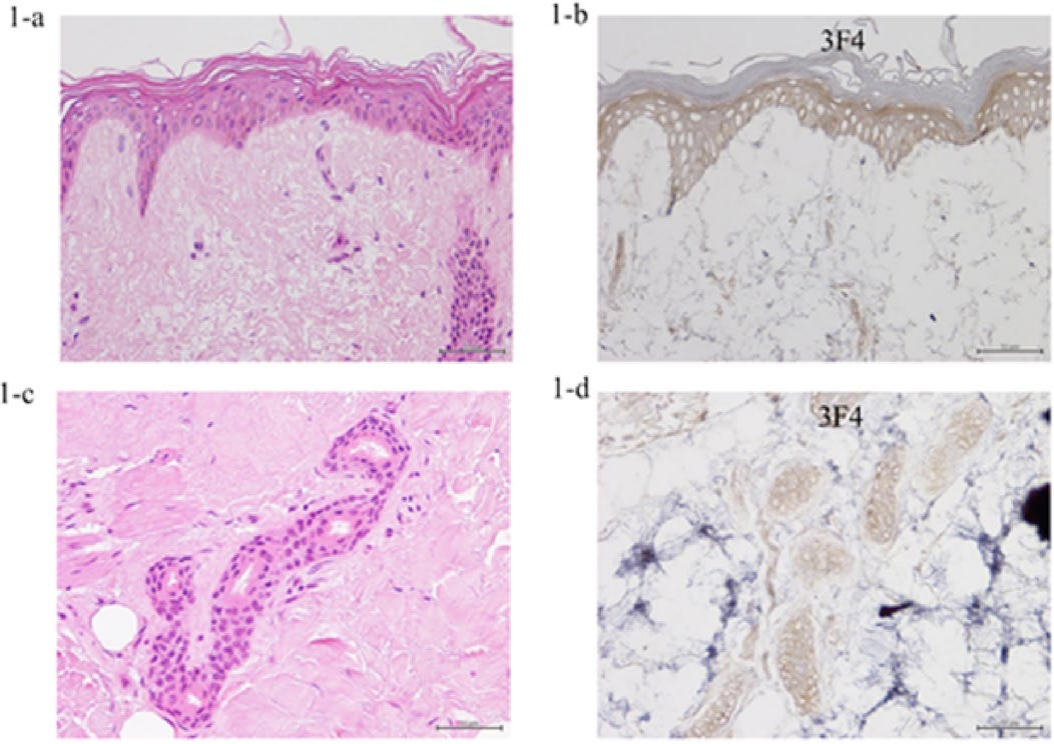
Pathological scalp findings in a patient with human prion disease (HPD). Hematoxylin–eosin staining (**1-a**, **1-c**); anti-prion protein (PrP) immunostaining using 3F4 antibodies (**1-b**, **1-d**) in the scalp of an HPD patient (Patient Number: #1 in Table 1). The presence of many sweat glands results in the accumulation of abnormal prion proteins in scalp tissue.

Neuropathological examinations were performed on HE-stained samples from an MM1 patient, subjected to immunohistochemical analysis using anti-PrP antibodies (antibody 3F4) (Figure 1). Keratinocytes and the nuclear layer were moderately stained with PrP immunostaining fluid. Dermis layer granulation tissue containing hair follicles was moderately stained by prion immunostaining (Figure 1). Hair follicles, granulation tissue, and keratinocytes that contained abnormal prion proteins were identified within the dermis layer.

### Sensitivity of the revised 2nd-generation RT-QuIC assay by the number of hair roots in autopsy cases (18 patients) with HPD

The 1st-generation RT-QuIC assay did not detect a positive reaction in the hair roots of patients with sporadic HPD. Consequently, we developed a revised 2nd-generation RT-QuIC assay to examine these patients’ hair roots (Supplementary Fig. S3 online). We used the revised 2nd-generation RT-QuIC assay to determine the PSA in hair samples of 18 patients, including hair (without roots) and hair root samples (14 MM1, 2 MM1 + 2C, and 2 MM2T cases) (Nos. 1–18; Table 2).

**Table 2:** Sensitivity of rooted and nonrooted hair RT-QuIC assays in autopsy cases of prion disease.

Hair with roots		Hair without roots	
Number of hair with roots	Sensitivity (%) (number)	Number of hair with no roots	Sensitivity (%) (number)
10–11	44.4 (n = 18)	10–11	0 (n = 18)
12–14	80 (n = 15)	12–14	0 (n = 15)
15–19	100 (n = 16)	15–19	0 (n = 16)
20–24	100 (n = 16)	20–24	6.2 (n = 16)
≥ 25	100 (n = 5)	≥ 25	0 (n = 5)

The revised 2nd-generation RT-QuIC assay was used to determine PSA in hair samples from 18 patients, including hair (without roots) and hair root samples (14 MM1, 2 MM1 + 2C, and 2 MM2T cases) (Nos. 1–18; Table 2).

Each patient was tested by comparing the RT-QuIC results from 10, 12, 15, 17, 20, and 25 hair roots and samples (without roots). The results revealed that <14 hair roots exhibited <80% positivity for the RT-QuIC reaction, and <10 hair roots exhibited 40% positivity. Importantly, all patients showed a positive (100%) RT-QuIC result when more than 15 hair roots were included. Thus, more than 15 fully intact hair roots should be in good condition to ensure a 100% positive RT-QuIC result. For hair samples (without hair roots), 6.2% (20 hair samples) were positive for the RT-QuIC reaction.

### Investigation of RT-QuIC conditions with different skin-lysing agents

We employed various skin lysates (as illustrated in Supplementary Fig. S3 online) due to the inability to achieve complete lysis of the scalp for the RT-QuIC assay. Despite evaluating multiple skin lysate preparations, none proved suitable for use in the RT-QuIC assay. Consequently, we proceeded with homogenizing approximately 90% of the skin, acknowledging that complete dissolution was unattainable. The composition of the skin-dissolving agents is detailed in S4 of the Supplementary Appendix

### Sensitivity and specificity of biomarkers (CSF biomarkers, including 14-3-3 protein, t-tau protein, RT-QuIC assay, and MRI findings) and hair roots from the prospective study

437 patients provided written informed consent to participate in our study. All participants provided their attending physicians with hair roots, CSF, MRI images, and the patient’s demographic information. In total, 125 of the 437 patients with <15 hair follicles were excluded from the study. Of the remaining 212 cases, we excluded those who refused to participate in the Surveillance Committee’s investigation and those undergoing follow-up or additional tests to receive an accurate diagnosis (n = 8).

We analyzed the CSF samples and hair roots of 177 HPD and 123 non-HPD patients. Table 3 shows that the sensitivity and specificity of t-tau protein in CSF were 86.4% (95% confidence interval (CI): 80.6%, 90.7%) and 65.0% (95% CI 44.9%, 81.2%), respectively. Similarly, the sensitivity and specificity of 14-3-3 protein were 83.6% (95% CI 77.5%, 88.3%) and 65.0% (95% CI 44.9%, 81.2%), respectively. In contrast, the RT-QuIC CSF assay (hu-1st-generation and 2nd-generation QuIC assay) had a 74.6% (95% CI 67.7%, 80.4%) sensitivity and 100% specificity, whereas the RT-QuIC hair root assay (revised 2nd generation QuIC assay) had a 52.5% (95% CI 45.2%, 59.8%) sensitivity and 100% specificity.

**Table 3:** Prospectively determined sensitivity and the specificity of CSF biomarkers, hair root QuIC assay, and DWI-MRI.

		CSF			Hair root	DWI-MRI
	14–3-3 protein	Total tau protein	hu-1st–generation RT-QuIC	2nd-generation RT-QuIC	RT-QuIC	
Sensitivity	83.6	86.4	74.6	74.6	52.5	100
Specificity	65.0	65.0	100	100	100	56.1

CSF, cerebrospinal fluid; QuIC, quaking-induced conversion; RT-QuIC, real-time quaking-induced conversion (hu-1st-generation and 2nd-generation QuIC assay); DWI-MRI, diffusion-weighted image on MRI. DWI-MRI findings were divided into abnormal (hyperintense areas in cortical lesions and/or basal ganglia) and normal findings. We defined at least three cortical areas and/or basal ganglia as positive MRI. A total of 300 patients were included in this prospective study: 177 patients with human prion disease and 23 without. Data are presented as n (%) unless otherwise indicated.

As shown in Supplementary Table S2 online, we determined the sensitivity of the CSF biomarkers and hair roots using RT-QuIC in patients with HPD. For the 139 sporadic HPD cases, the CSF was positive for t-tau and 14-3-3 proteins, showing 89.2% and 86.3% positivity, respectively. The positivity of the CSF (hu-1st-generation QuIC assay and 2nd-generation QuIC assay) and hair root RT-QuIC assays (revised 2nd-generation QuIC assay) were 85.6% and 56.1%, respectively. In 37 cases of genetic HPD, 26 were positive for 14-3-3 protein, and 28 were positive for t-tau protein. The positivity of RT-QuIC was 37.8% for hair roots and 32.4% for the CSF. We included one case of acquired HPD, which showed a 100% positive rate with the CSF and hair root RT-QuIC assays. The sensitivity of DWI-MRI was 100%, and the specificity was 56.1% (Supplementary Table S2 and S3 online).

We included one definite case of V180I genetic CJD (gCJD). This patient’s test results were positive for the hair root RT-QuIC assay, t-tau protein, and 14-3-3 protein but negative for the CSF RT-QuIC assay (Supplementary Table S2 and S4 online). The patient’s CSF RT-QuIC assay had a sensitivity of 4.5% and a sensitivity of 13.6% for the hair root RT-QuIC assay (Supplementary Table S3 online).

Out of 177 HPD patients, 139 were sporadic-, 37 were genetic-, and one was acquired-type HPD (Supplementary Table S2 online). All cases exhibited typical findings on DWI-MRI. The sensitivities for the t-tau, 14-3-3 proteins, and the RT-QuIC assay in the CSF were 86.3%, 89.2%, and 85.6% for sporadic-type HPD, 70.3%, 75.7%, and 32.4% for genetic-type HPD, and 100% for all cases with acquired-type HPD, respectively. However, in hair root samples, the sensitivities of the RT-QuIC assay were 56.1%, 37.8%, and 100% for the sporadic-, genetic-, and acquired-type HPD, respectively.

The sensitivity for the genetic-type HPD was lower than that for other varieties. Thus, CSF biomarkers and RT-QuIC hair root assay were reanalyzed for all types of genetic HPD (Supplementary Table S3 online). The biomarker sensitivities for V180I, E200K, and V180I + M232R in gCJD were higher than those for GSS (P102L) and octapeptide repeat insertion. However, the RT-QuIC assay sensitivity for V180I was lower than that for the sporadic and genetic types (E200K and M232R).

Our prospective study included 123 non-HPD patients with the following diagnostic distribution: epilepsy, 33 patients (26.8%); hypoxic-ischemic encephalopathy, 23 patients (18.7%); metabolic encephalopathy, 21 patients (17.1%); autoimmune encephalitis/encephalopathy, 28 patients (22.8%); viral encephalitis, 12 patients (9.8%); and cerebrovascular accident, 6 patients (4.9%).

## Discussion

We implemented scalp sampling methodology based on Zhang et al.’s demonstration that scalp tissue exhibits the highest sensitivity for detecting skin PSA compared to other anatomical sites^13,14^.

We developed and established a first-generation scalp and hair root RT-QuIC assay with a revised second-generation RT-QuIC assay. RT-QuIC assays on 22 scalp and hair root samples (including ≥15 hair roots) from 22 HPD patients revealed 100% positivity. We evaluated the distribution of PSA across the various scalp regions and found the average log SD50 in the three layers to be the same level in HPD patients (Supplementary Fig. S3 online and Supplementary Tables S4 and S5 online).

All scalp samples were collected at autopsy from patients several years post disease onset. Given that direct scalp biopsy would cause significant patient discomfort, we are developing a noninvasive collection method using post-bath brushing to obtain samples from living patients. This approach will enable investigation of RT-QuIC positivity kinetics throughout disease progression in future prospective studies.

We investigated methods for dissolving the scalp tissue to further validate using the RT-QuIC assay for scalp samples. We used different skin-lysing agents (Supplementary Fig. S4 online) because we could not lyse the scalp for the RT-QuIC assay. Our novel method involved washing the scalp several times with phosphate-buffered saline instead of one multibead shocker, as the components of the skin-lysing agents may inhibit the RT-QuIC reaction. The skin was cut into shreds under a stereomicroscope and homogenized electrically under visual observation (Supplementary Fig. S3 online). Pathologically abnormal prion proteins in the scalp were revealed by immunostaining (Figure 1). Our findings the RT-QuIC assay of scalp samples may offer diagnostic potential for prion diseases. However, comprehensive validation studies involving larger patient cohorts and appropriately provide preliminary evidence that matched controls are essential before considering clinical implementation as an alternative to brain biopsy.

We used the RT-QuIC assay to identify positive reactions in scalp tissue samples from patients with HPD. However, the sensitivity of the RT-QuIC assay to detect a positive reaction in the hair root was low in the first-generation RT-QuIC. Thus, we emphasized the temperature and number of hair roots in our investigation (Supplementary Fig. S5 online). As shown in Supplementary Fig. S5 online, the ideal temperature was 55°C, and—as demonstrated in Table 2—the ideal number of hair roots was 15. However, the sensitivity of hair without hair roots was quite low. The revised 2nd-generation RT-QuIC assay was used to define these circumstances.

Our prospective study used the second-generation RT-QuIC assay to analyze hair root samples from patients with HPD. The sensitivity and specificity of the RT-QuIC assay were 52.5% and 100%, respectively. Sporadic-type HPD showed a higher RT-QuIC assay sensitivity than the genetic-type HPD (Supplementary Table S3 online). The extremely low sensitivity for identifying V180I hereditary HPD may be responsible for these results.

The scalp SD50 was lowest in V180I cases and approximately 6–8 times lower than the SD50 of MM1 and MM2 cases. Previous studies indicated that immunoreactivity to PrP^Sc^ is very weak in V180I-gCJD patients^15–21^. The low accumulation of PrP^Sc^ in the brain may justify the low positivity of the RT-QuIC assays^22^.

For optimal outcomes, the following must occur: (1) the hair sample must include the root; (2) there should be at least 15 hair roots in the sample; and (3) the updated second-generation RT-QuIC assay should be used.

Patients who refuse (or cannot tolerate) CSF testing, including those with severe back deformities or too much myoclonus, may agree to test with the RT-QuIC assay as it is a noninvasive and relatively sensitive option. The sensitivity of the hair root RT-QuIC can be increased to 70% when testing is limited to those patients with at least 15 hair follicles that have been washed, cleaned, and newly brushed (Supplementary Table S6 online).

In sporadic Creutzfeldt–Jakob disease (CJD), the PRNP gene polymorphism at codon 129 typically exhibits a distribution of 97% Met/Met (MM), 3% Met/Val (MV), and 0.5% Val/Val (VV). The frequency of VV and MV in 129 polymorphisms of *PRNP* in a three-year study of this size is estimated to be 3% of all cases. However, the actual number is 6 for MV, 1 for VV, and 122 for MM, based on statistical trends. However, all patients in this study had the MM genotype. It is important to closely examine how the hair root QuIC assay performs in sCJD patients with MV and VV variants at codon 129. An effective assay could be a game-changer for early detection and provide crucial insights into how these polymorphisms affect prion pathology.

We acknowledge that our study predominantly focused on the MM genotype of the PRNP gene codon 129 polymorphism. This distribution reflects the actual epidemiological profile in the Japanese population. Recent data demonstrate that the genotype frequencies among Japanese patients with sporadic CJD are distributed as follows: MM (96.5%), MV (3.0%), and VV (0.5%)^23^. Therefore, research on PRNP codon 129 polymorphism is inherently skewed toward the MM genotype, which accurately represents the predominant variant in our target population.

This study has some limitations that should be considered. First, all cases exhibited the MM genotype at codon 129 of the PRNP gene, which reflects the genetic characteristics of the Japanese population and limits the generalizability of our findings to populations with different genotype distributions.

Previous studies have shown that skin samples from patients with CJD can transmit infection in experimental mouse models; however, the current observation period may have been insufficient to establish infectivity in such models.

Second, our use of human- and hamster-derived recombinant prion proteins for the RT-QuIC assay may affect reproducibility across international settings, as the production of human-derived recombinant proteins is not feasible in many countries. Third, our patient cohort was drawn from a prospective study on rapidly progressive dementia, with patients referred after undergoing blood tests, CSF analysis, and MRI. This introduces a potential referral bias and may limit the representativeness of the study population relative to all suspected prion disease cases. Blood neurofilament light chain (NfL) is a promising marker in prion diseases, particularly for early screening, prognosis, and presymptomatic monitoring. Research on blood biomarkers is also rapidly progressing in the field of rapidly progressive dementias. NfL may also aid in the evaluation of disease severity and treatment response in other neurodegenerative disorders and autoimmune encephalitis^24–26^. However, current evidence remains limited, with sensitivity and specificity still insufficient, underscoring the need for large-scale longitudinal validation. Fourth, the study was conducted from a clinical perspective and incorporated multiple biomarkers, including α-synuclein. This integrative approach may have introduced clinical judgment bias in patient selection and result interpretation. Fifth, although the assay demonstrated acceptable specificity, its sensitivity was limited to approximately 50%, which is considerably lower than that of the CSF-based RT-QuIC assay. This reduced sensitivity may be attributed to the small amount of scalp tissue obtained through post-shampoo brushing, which likely resulted in low prion protein concentrations. Additional limitations include the absence of standardized sampling protocols, variability in sample collection timing relative to disease progression, and a lack of external validation across laboratories. Collectively, these factors constrain the immediate clinical applicability of the scalp-based RT-QuIC assay and underscore the need for larger, multicenter validation studies.

In conclusion, our novel method uses a relatively small scalp tissue sample, second-generation RT-QuIC assays, and minimally invasive techniques (such as collecting hair and hair roots from the pillow, after brushing and bathing, tapping from the brush or desk) to obtain hair root samples and clinically meaningful results. Our novel method could emerge as an effective tool, paving the way for further development of novel methods for diagnosing neurodegenerative diseases.

## Methods

### 4.1 Summary of HPD profiles in autopsy

All patient information was obtained using a standardized form (Supplementary Fig. S1 online). Patients’ demographic information was obtained from the Japan Prion Disease Surveillance Committee (J-PrD-SC) and the institutions where autopsies were conducted. Table 1 shows that the endpoint RT-QuIC assay by hu-1st generation was implemented to test nine “native” brain samples from MM1 sCJD patients on Parchi’s classification (patient No. 1–8) and 22 “native” scalp samples from 22 patients with confirmed HPD, including 14 cases of MM1 (Nos. 1–14), two cases of MM1 + 2C (Nos. 15–16), two cases of MM2T (Nos. 17–18), and four cases of V180I (Nos. 19–22). We defined “native” as freshly collected scalp specimens without formalin and formic acid treatment

Parchi’s classification of six distinct sCJD phenotypes is based on three key criteria: the molecular size of the protease-resistant prion protein as determined by brain tissue western blotting, the polymorphism at codon 129 of the *prion protein gene (PRNP)*, and characteristic neuropathological findings. We utilized these criteria to systematically categorize sCJD cases in our study.

We analyzed the PSA in the epidermis, dermis, and hypodermis of MM1 (n = 6) (No. 1–6), MM1 + 2C (n = 1) (No. 15), and MM2T (n = 1) (No. 17) cases, using the endpoint RT-QuIC assay. This analysis provided information on prion distributions across different scalp layers. This analysis was also performed in four cases of V180I samples (Nos. 19–22) (Table 1). Five human scalp samples obtained from non-HPD patients (patients with dementia and Lewy body disease) were used as negative controls.

### Scalp sample collection methods

We implemented two distinct approaches for obtaining the scalp samples. In instances where autopsies were performed, a punch biopsy on the scalp was performed. This involved the insertion of a cylindrical punch (5 mm diameter) into the deep dermis or subcutaneous tissue, followed by extraction of a hollowed-out specimen cut at the base. All sampling sites were uniformly sampled as parietal lobes. We collected several samples of autopsy-derived scalp using punch biopsy. One sample was left intact without separation and analyzed. The remaining samples were separated under a stereomicroscope into dermis, epidermis, and subcutaneous tissue and each layer was analyzed.

All sampling sites were uniformly sampled as parietal lobes. We collected several samples of autopsy-derived scalp using punch biopsy. One sample was left intact without separation and analyzed. The remaining samples were separated into three layers under stereomicroscope and each layer was analyzed.

For patients diagnosed with prion diseases, a minimally invasive technique was employed. It included collecting hair and hair roots from pillows, after hair brushing, after bathing, and tapping hair from brushes or desks. We recommend sampling the scalp and hair follicles using a hairbrush after bathing to enhance detection sensitivity

### Neuropathological examination

All postmortem investigations were conducted within 22.5 h of death. The brain and upper cervical spinal cord were fixed in neutral-buffered formalin (20%) for 4 weeks. Afterward, the resultant tissue blocks were immersed in formic acid (95%) for 1 h to deactivate prion infectivity. After embedding in paraffin, the specimens were cut into 9-μm-thick sections, deparaffinized in lemosol, rehydrated with an ethanol gradient, and stained. As a part of the neuropathological examination, sections were stained with hematoxylin–eosin (HE) staining^11^. Hydrolytic autoclaving for antigen retrieval was performed, followed by immunohistochemical analysis using a mouse monoclonal antibody against PrP (3F4, dilution 1:100; Dako, Glostrup, Denmark). PrP immunostaining was conducted as previously described^11^.

### Endpoint RT-QuIC assay

We used the endpoint RT-QuIC method with hu-1st-generation QuIC assay to quantitatively measure prion activity within the “native” brain and scalp tissue samples. The RT-QuIC reaction was performed as previously described (Supplementary Appendix)^5^. The Spearman–Kärber method calculates the 50% seeding dose (SD50) in nativebrain tissue samples and scalps^27^. A positive reaction was defined by the RT-QuIC assay, as described in the Methods section of the Supplementary Appendix.

### Revised 2nd generation QuIC assay suitability for use with hair and hair root

We attempted to stimulate prion activity from hair and hair follicles using the RT-QuIC assay. The RT-QuIC assay was performed using at least 25 hair roots and hair collected from pathological scalp samples. We attempted the RT-QuIC assay with >1000 modifications from the conditions of the hu-1st generation QuIC assay, but were not successful. Because the 2nd generation QuIC assay was unsuccessful, we utilized a revised 2nd generation QuIC assay changing the species of recombinant prion protein (human and bank vole). Notably, the revised 2nd generation QuIC assay with human prion protein was successful.

To determine the criteria to achieve 100% specificity, we conducted a study involving 123 participants: 66 healthy individuals, 40 diagnosed with Alzheimer’s disease, and 17 diagnosed with Lewy body disease. All participants provided a minimum of 15 hair follicles.

### Prospective study patients

During the three-year prospective study (October 2019–March 2022), CSF and hair root biomarkers were analyzed at Nagasaki University using QuIC assays from 337 patients with rapidly progressing dementia (Supplementary Fig. S2 online and Supplementary Table S2 online). Of these, 208 cases provided more than 15 hair with root samples, CSF samples, complete patient information data, and complete MRI data were included. We obtained written informed consent from 300 cases for CSF analysis and revised 2nd RT-QuIC hair root assays. Eight cases were excluded because they refused further participation and/or had insufficient clinical information.

J-PrD-SC all patients’ diagnoses and subsequent analysis results. Genomic DNA extracted from peripheral blood leukocytes was used for PrP genotyping. Diagnosis and categories for all cases were determined according to previous studies and the WHO diagnostic criteria^6,28,29^. The ethics committee at Nagasaki University Graduate School of Biomedical Sciences endorsed the study protocol (ID Nos. UMIN000038398, UMIN000016855, and UMIN000003301). All case data were collected for a prospective epidemiological study on HPD diagnosis and surveillance, with diagnosis defined as per previously published protocols^29,30^.

### Biochemical analysis of CSF samples

ELISA was used to measure t-tau protein in the CSF according to the manufacturer’s instructions for all experiments. As previously described, we measured 14-3-3 protein levels from all human CSF samples using western blotting for 14-3-3 protein^31,32^. The RT-QuIC assay was performed using recombinant human PrP (hu-1st-generation QuIC assay)^5^, and recombinant hamster PrP (2nd -generation QuIC assay). CSF samples collected from all patients with suspected HPD were stored in aliquots at −80°C. All assays were repeated once every 2 weeks from 2011 to 2020 to avoid repeated freeze–thaw cycles.

### MRI findings

Deidentified DWI, b0, and fluid-attenuated inversion recovery images were converted to the Digital Imaging and Communication in Medicine (DICOM®) format before the observer study^32–34,35^. All MRI images were obtained from the participating hospitals using 1.5- or 3.0-Tesla scanners. MRI images of atypical HPD cases were assessed by neuroradiologists from Tokushima University and J-PrD-SC. In addition to analyzing the DWI, we confirmed the ADC map.

### Statistical analysis

SPSS version 24 and R analysis software were used for statistical analysis.

In this study, we used this software only for the calculation of sensitivity and specificity with respect to statistics.

## Supplementary Information

Below is the link to the electronic supplementary material.

Supplementary Material 1.

## Data Availability

All data generated or analyzed during this study will be made publicly available through our dedicated website [https://www2.am.nagasaki-u.ac.jp/prion-cjd/research/kokusai-ishi.html]. This website has been specifically prepared to ensure comprehensive access to the dataset for all researchers and interested parties following the publication of this manuscript.

## Declarations

### Statements from author and publisher

We obtained all authors’ consent for publication.
