# Supplementary material for: A noninvasive test for human prion disease using hair roots and scalp
Source: Sci Rep. 2025 Nov 24;15:41492. doi: 10.1038/s41598-025-25310-y (PMC12644664; doi:10.1038/s41598-025-25310-y)
Supplement: Supplementary file 1 — Supplementary Material 1. [file 41598_2025_25310_MOESM1_ESM.docx]

**Supplementary Material**

**A noninvasive test for human prion disease using hair roots and scalp**

**This appendix provides readers with additional information about this study.**

**Table of Contents**

Authors and their contributions P1

A detailed method of real-time quaking-induced conversion (RT-QuIC) assay using human materials P3

Supplementary Fig. S1 online. Patient profiles and information: Prion Disease Surveillance Study P5

Supplementary Fig. S2 online. The study participant selection process P7

Supplementary Fig. S3 online. RT-QuIC response determined from phosphate-buffered saline (PBS) and radioimmunoprecipitation assay buffer (RIPA) buffers P10

Supplementary Fig. S4 online. Prion seeding distribution activity across various scalp regions P12

Supplementary Fig. S5 online. RT-QuIC reactions at different temperatures P14

Supplementary Table S1 online. Sensitivity and specificity of skin RT-QuIC assays in previous reports P16

Supplementary Table S2 online. The sensitivity of cerebrospinal fluid (CSF) biomarkers and the hair root QuIC assay in human patients with prion disease P18

Supplementary Table S3 online. The sensitivity of CSF biomarkers and the hair root QuIC assay in genetic prion disease P19

Supplementary Table S4 online. Biomarker and the hair root QuIC assay sensitivity in definite cases of human prion disease P20

Supplementary Table S5 online. Endpoint RT-QuIC assay in native scalp layers obtained from human patients with prion disease P21

Supplementary Table S6 online. The sensitivity of hair root RT-QuIC measures in patients with human prion disease (HPD) unable to undergo CSF testing P22

**Authors and their contributions**

Thi-Thu-Trang Dong ^1^ M.D., Hiroyuki Honda ^2^ M.D., Akio Akagi ^3^ M.D., Yasushi Iwasaki ^3,4^ M.D., Hitaru Kishida ^5,7^ M.D., Tadashi Tsukamoto ^4,6,7^ M.D., Kensaku Kasuga ^7, 8^ M.D., Hirotsugu Takashima ^9, 10^ M.D., Tatsuhiro Terada ^9, 10^ M.D., Kensuke Ikenaka ^7,11^ M.D., Takeshi Ikeuchi ^8^ M.D., Tsuyoshi Mori ^12^ M.D., Hideki Mochizuki ^7, 11^ M.D., Kenjiro Ono ^13^ M.D., Yoshihisa Takiyama ^14,15^ M.D., Tsuyoshi Hamaguchi ^4, 7, 16^ M.D., Hiroyuki Murota ^17^ M.D., Nobuo Sanjo ^4, 7,18^ M.D., Takeshi Fujimoto ^19^ M.D., Michio Kitayama ^20^ M.D., Koji Fujita ^7,21^ M.D., Motohiro Yukitake ^4, 22^ M.D., Shinsuke Fujioka ^23^ M.D., Noriyuki Nishida ^24^ M.D., Yoshio Tsuboi ^4,22^ M.D., Tetsuyuki Kitamoto ^4, 7, 25^ M.D., Masaki Takao ^4, 6^ M.D., Masahito Yamada ^4, 7, 26^ M.D., Hidehiro Mizusawa ^4,6,7^ M.D., Katsuya Satoh ^4,7, 27, 28^ M.D

^1^ Outpatient Clinic for Senior Staff, 108 Military Central Hospital, Hanoi, Vietnam

^2^ Neuropathology Center, Department of Neurology, National Hospital Organization, Omuta National Hospital, Japan

^3^ Department of Neuropathology, Institute for Medical Science of Aging, Aichi Medical University, Japan

^4^ Research Committee of Prion Disease and Slow Virus Infection, Research on Policy Planning and Evaluation for Rare and Intractable Diseases, Japan

^5^ Department of Neurology, Yokohama City University Medical Center, Yokohama, Japan

^6^ National Center of Neurology and Psychiatry (NCNP), Japan

^7^ Japan Prion Disease Surveillance Committee (PrD-SC), Japan

^8^ Department of Molecular Genetics, Brain Research Institute, Niigata University, Japan

^9^ Department of Neurology, National Epilepsy Center, NHO Shizuoka Institute of Epilepsy and Neurological Disorders (NEC), Japan

^10^ Department of Biofunctional Imaging, Preeminent Medical Photonics Education & Research Center, Hamamatsu University School of Medicine, Japan

^11^ Department of Neurology, Osaka University Graduate School of Medicine, Japan

^12^ Division of Microbiology, Department of Infectious Diseases, Faculty of Medicine, University of Miyazaki, Japan

^13^ Department of Neurology, Graduate School of Medical Sciences, Kanazawa University, Japan

^14^ Department of Neurology, Fuefuki Central Hospital, Japan

^15^ Department of Neurology, Graduate School of Medical Sciences, University of Yamanashi, Japan

^16^ Department of Neurology, Kanazawa Medical University, Uchinada, Japan

^17^ Department of Dermatology, Nagasaki University Graduate School of Biomedical Sciences, Nagasaki, Japan

^18^ Department of Neurology and Neurological Science, Graduate School, Tokyo Medical and Dental University, Tokyo, Japan

^19^ Department of Neurology, Sasebo City General Hospital, Japan

^20^ Department of Internal Medicine, Kawasaki Medical School General Medical Center

^21^ Department of Neurology, Tokushima University Graduate School of Biomedical Sciences, Japan

^22^ International University of Health and Welfare, Ookawa, Japan

^23^ Department of Neurology, Faculty of Medicine, Fukuoka University, Fukuoka, Japan

^24^ Department of Molecular Microbiology and Immunology, Nagasaki University Graduate School of Biomedical Sciences, Japan

^25^ Department of Prion Protein Research, Division of CJD Science and Technology, Tohoku University Graduate School of Medicine, Sendai, Japan

^26^ Department of Internal Medicine, Division of Neurology, Kudanzaka Hospital, Tokyo, Japan

^27^ Department of Health Sciences, Unit of Medical and Dental Sciences, Nagasaki University Graduate School of Biomedical Sciences, Japan

^28^ Department of Brain Research Unit, Leading Medical Research Core Unit, Nagasaki University Graduate School of Biomedical Sciences, Japan

**Authors’ contributions**

Wrote the manuscript: TTTD, NN, and KS. All authors provided feedback on the manuscript.

Neuropathological analysis: HH, AA, YI, TI, TK, and MT.

Samples provided (scalp and hair root) and research participation: AA, YI, and MT.

Samples provided (scalp) and research participation: HK, TT, KK, HT, TT, KI, TI, TM, HM, KO, YT, TH, NS, TF, MK, KF, MY, SF, and YT.

CSF testing: KS.

DNA analysis and typing of PrP^Sc^ in human prion disease: TK.

MRI analysis: KF.

Statistics, statistical calculations, and database creation: TTTD and KS.

Japan Prion Disease Surveillance Committee member, from 2011 till 2022: HK, TT, KK, KI, HM, YT, FT, TH, NS, KF, TK, MY, HM, and KS.

**A detailed method of real-time quaking-induced conversion (RT-QuIC) assay using human materials**

**Hu-1^st^ generation RT-QuIC assay investigation**

This study’s RT-QuIC assays and endpoint RT-QuIC assay analysis were similar to previously described unfixed brain RT-QuIC assays.^1^ The RT-QuIC reaction mix was composed of 50 mM PIPES (pH: 7.0), 500 mM NaCl, 10 µM Thioflavin T (ThT), 0.1 mM ethylenediaminetetraacetic acid tetrasodium salt hydrate (EDTA), and recombinant human PrP (residues: 23–231 at 129 M) in the first-generation and revised second‑generation RT-QuIC assays.

Reaction mix aliquots (90 µl) were mixed with 10 µl of human samples and loaded into a 96-well black plate with a clear bottom (Nunc 96 well; Sigma–Aldrich, USA). We measured 4–8 replicates of each diluted sample and monitored PrP amyloid formation for 48 h. We used a FLUOstar OMEGA plate reader (BMG Labtech, Germany) to seal and incubate the plates at 55°C with intermittent shaking cycles (60 s in double-orbital motion at 700 rpm) and rest (60 s). ThT fluorescence readings were made at the 450 ± 10 nm (excitation) and 480 ± 10 nm (emission) wavelengths every 45 min. The 50% seeding dose (SD50) was calculated in unfixed, formalin-fixed, and formic acid-treated brain tissue using the Spearman–Kärber method. The RT-QuIC assay was considered positive if: (1) the absorbance increased within 150 cycles and the levels remained stable after reaching the maximum value; (2) the maximum absorbance was >6 times higher than the initial absorbance; and (3) only recombinant protein was used to maintain the lot quality when the prepared recombinant protein showed an SD50 of ≥10^7^ by the RT-QuIC assay in the brains of MM1-type sCJD patients. The assay was repeated at least twice, and Spearman–Kärber analysis was used to estimate the seeding dose (SD50). The SD50 was calculated as: xp = 1 + 1/2d − dgp, where xp = 1 is the highest log dilution giving all positive responses, d is the log dilution factor, p is the proportion of positive reaction at a given dose, and gp is the sum of values of p for xp = 1 and all higher dilutions.^2^

The second-generation RT-QuIC assay contained 10 mM phosphate buffer at pH 7.4, 1 mM EDTA at pH 8.0, 300 mM NaCl, 10 μM ThT, 0.002% SDS, and 0.1 mg/mL recombinant Syrian hamster prion protein (Ha rPrP 90–231). A FLUOstar OMEGA plate reader (BMG Labtech, Germany) was used to incubate the plates at 55°C, with ThT fluorescence collected every 45 min using 450 ± 10 nm (excitation) and 480 ± 10 nm (emission) wavelengths. Furthermore, the Bank vole (Bv) RT-QuIC reaction buffer comprised 10 mM of phosphate buffer (pH: 7.4), 300 mM NaCl, 0.1 mg/mL Bv rPrP (aa residues: 23–231, with methionine at codon 129), 10 μM ThT, and 10 μM EDTA.

**References**

1. Atarashi R, Satoh K, Sano K, et al. Ultrasensitive human prion detection in cerebrospinal fluid by real-time quaking-induced conversion. *Nat Med.* 2011; 17(2): 175–78.

2. Takatsuki H, Satoh K, Sano K, et al. Rapid and quantitative assay of amyloid-seeding activity in human brains affected with prion diseases. *PLOS ONE.* 2015; 10(6): e0126930.

**Preparation of brain tissue homogenate**

Brain tissue testing used the RT-QuIC assay to determine the SD50. Single-use disposable tubes and beads were used to avoid contaminating the tissue samples, and the processes were carried out on different days. A multibead shocker (Yasui Kikai, Osaka, Japan) was used to homogenize the brain tissue samples in 10% (weight/volume) ice-cold phosphate-buffered saline supplemented with a protease inhibitor mixture (Roche, Mannheim, Germany). The samples were centrifuged at 6,000 rpm for 2 min and stored at −80°C.

**Scalp tissues and preparation**

We implemented two distinct approaches to obtain scalp samples. For autopsies, a punch biopsy of the scalp was performed. This involved inserting a cylindrical punch (5 mm diameter) into the deep dermis or subcutaneous tissue and then extracting a hollowed-out specimen cut at the base. All sampling sites were uniformly sampled as parietal lobes. We collected several samples of the autopsy-derived scalp by punch biopsy. One sample was left intact without separation and analyzed. The remaining samples were separated under a stereomicroscope into dermis, epidermis, and subcutaneous tissue, and each layer was then analyzed.

The autopsy process was designed to ensure that cross-contamination did not occur between the scalp and brain tissues and the cadavers. The scalp tissues were washed thrice in 1 × phosphate-buffered saline (PBS) and minced. A Power Masher 2 machine was used to homogenize scalp tissue samples in 10% (weight/volume) ice-cold PBS supplemented with a protease inhibitor mixture (Roche, Mannheim, Germany) for 2–4 min. The samples were centrifuged at 6,000 rpm for 2 min and stored at −80°C.

**Root hair samples**

After the scalp punch biopsy, hair and hair follicles were harvested from the remaining tissue using tweezers. We used endpoint RT-QuIC assays to evaluate the 18 samples (approximately 5–15 mm). These included samples of hair roots and hair (without roots) from 18 definite cases of human prion disease (HPD). These included 12 MM1, 1 MM2T, 1 MM1 + 2C, and 2 V180I cases. Overall, 10 root hairs from 18 cases, 12 from 4 cases, 15 and 20 from 16 cases, and 25 from 5 cases were used for each sample in the RT-QuIC assay. The same number of root hairs from the control group was used in the experiment. Five root hair specimens from healthy subjects were used as negative controls. SD50 was calculated using the Spearman−Kärber method.

**Hair sample preparation**

The hair homogenates were placed in PBS (10–25 hair roots/120–200 μl PBS) after being washed three times in 100–200 μl PBS and cut into short pieces. The hair homogenate was prepared by mashing short hair pieces for 2–4 min with a Power Masher 2 machine in ice-cold PBS. The samples were centrifuged for 5 min at 6,000 rpm at 4°C to produce the supernatant (HS1). HS1 was stored at −80°C before performing the RT-QuIC analysis.

**Supplementary Fig. S1 online. Patients’ profiles and information (1): The Prion Disease Surveillance Study**

**Patients’ profiles and information (2): The Prion Disease Surveillance Study**


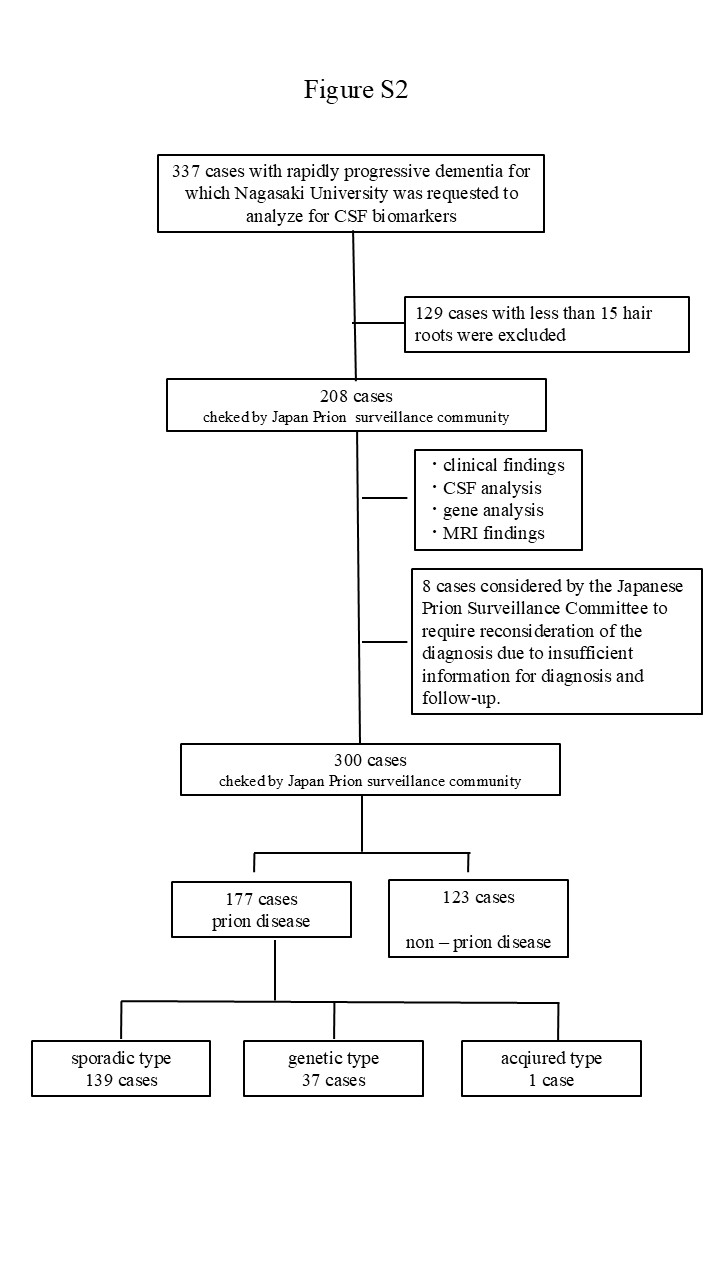
 Supplementary Fig. S2 online. **The study participant selection process**

1) First contact and check

- From 2019 to 2022, we offered no-cost testing to any patient with rapidly progressive dementia who requested it while being treated at Japanese hospitals or clinics for suspected or potential HPD. Additionally, study participants received free testing involving novel hair root testing methods.
- Patients who provided written informed consent for the Japan Prion Disease Surveillance Committee (J-PrD-SC) testing and biomarker measurements were examined by doctors at each participating hospital in Japan.
- Each hospital or clinic in Japan that signed up to measure biomarkers delivered their CSF testing samples at −80°C to Nagasaki University.

2) First selection: exclusion criteria

- If the patient, their family, or physicians refused assessment by the J-PrD-SC, they were excluded from the study.
- Patients with insufficient clinical information and test results—including blood tests for diagnosis—were excluded from the study.
- Information on patients with suspected HPD was collected when the hospital or clinic’s doctor provided CSF samples and the patients’ information to Nagasaki University. Patients without complete information were excluded from the study.
- We also excluded patients whose genetic prion protein analysis was not performed for final diagnosis.

3) Second selection

Review of cases by the J‑PrD‑SC

The HPD surveillance by the J-PrD-SC started in April 1999 and covered 10 geographic areas in Japan from:

1. Each patient’s family registration with the Intractable Disease Treatment Research Program and the Ministry of Health, Labor and Welfare, Japan.
2. Notifications based on the Infectious Diseases Control Law in Japan.
3. Cases in which clinicians suspected prion disease and requested genetic testing from the J-PrD-SC genetic analysis team.
4. The case in which a clinician suspected prion disease and requested a CSF laboratory test from the J-PrD-SC CSF analysis team.

The J-PrD-SC comprises 20 members from 10 areas covering all regions of Japan. A neurologist in each area evaluated the patients and collected the necessary information, including medical and clinical history, neurological findings, and laboratory data (including CSF biomarkers*, MRI findings based on prion disease criteria**, electroencephalography (EEG) findings, and genotype data).

Meetings were held twice yearly on two consecutive days. All members were required to attend these meetings and examine cases from all perspectives to make a definitive diagnosis. Neuropathologists who were members of the J-PrD-SC presented neuropathological examination and western blotting results for PrP^Sc^.

If the information required for a case at the first meeting was insufficient, then a J-PrD-SC member requested additional information at a second meeting. The committee reassessed atypical cases that were difficult to diagnose at 6 or 12 months. At this point, the committee reviewed any changes in clinical symptoms and other test results.

CSF biomarkers*: total tau protein measured by ELISA (>1,300 pg/ml considered positive) and 14-3-3 protein assessed by western blotting and ELISA (if the western blot yields a poor signal and it is unclear whether the result is negative or weakly positive, confirm using ELISA [>10.000 SU/ml] and RT-QuIC assay (first- and second-generation assays).

MRI findings based on the prion disease criteria**: the radiological criteria for prion disease diagnosis required typical high signal intensity changes on fluid-attenuated inversion recovery (FLAIR) or diffusion-weighted imaging (DWI) in either two or more cerebral cortical regions (excluding the frontal lobe) or in both the caudate nucleus and putamen (corpus striatum).

- Patient classification and diagnosis

HPDs were classified into four types as follows:

1. sporadic (sCJD)
2. genetic (genetic CJD, GSS, FFI, and 96-bp insertion)
3. acquired (iatrogenic CJD or variant CJD)
4. unclassified

We excluded cases without DNA analysis from this study.

- Diagnostic criteria

1. Sporadic CJD was diagnosed according to the classic Master’s criteria.
2. Genetic prion disease was diagnosed according to neuropsychiatric findings compatible with prion disease.
3. Creutzfeldt–Jakob disease was diagnosed using the WHO criteria (2001).

* Patients with prion disease were categorized as (i) definite cases that were pathologically confirmed, (ii) probable cases, and (iii) possible cases according to the Masters’ criteria.

* To ensure accuracy, we excluded cases with missing information or other possible diagnostic variables.

**Supplementary Fig. S3 online.** **RT-QuIC response determined from phosphate-buffered saline (PBS) and radioimmunoprecipitation assay buffer (RIPA) buffers**
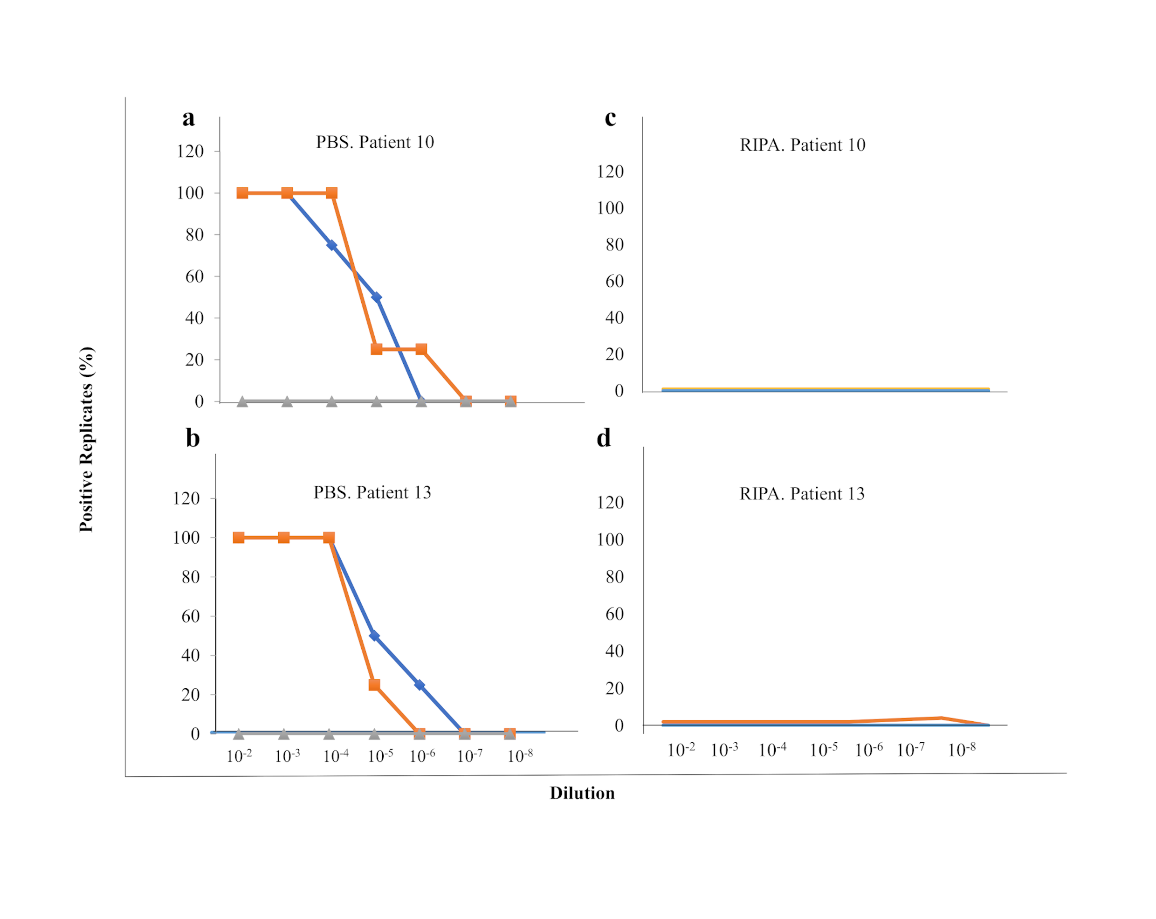


Quadruplicate RT-QuIC reaction samples were seeded with 10 µl of scalp tissue with serial 10^−2^–10^−8^ dilutions using PBS and RIPA buffer from two patients (patients 10 and 13). Negative control reaction samples were also seeded with a 10^−2^–10^−8^ dilution. Each trace represents the average normalized percent positive replicate (y-axis) of four replicate wells plotted as a function of the dilution (x-axis). Each assay was repeated ≥twice.

**Method**

Two scalp samples (approximately 40–50 mg and 3 × 5 mm each) were collected from two patients with HPD (two MM1 cases: patients 10 and 13). Scalp tissue samples were homogenized in 10% (weight/volume) PBS buffer for 2–4 min using a Power Masher 2 machine after being washed thrice in 1 × PBS and minced. The samples were centrifuged at 6,000 rpm for 2 min and stored at −80°C.

The scalp samples from patients 10 and 13 were soaked in a RIPA lysis buffer (50 mM Tris-HCl, pH 8.0, 150 mM sodium chloride, 0.5 w/v% sodium deoxycholate, 0.1 w/v% sodium dodecyl sulfate, 1.0 w/v% NP-40 substitute) and 3 ml of complete RIPA per gram of tissue for 30 min after mincing. The scalp tissue samples were homogenized with 10% (weight/volume) RIPA buffer using a Power Masher 2 machine for 2–4 min. Then, the samples were centrifuged at 6,000 rpm for 2 min. RT-QuIC was performed immediately after sample preparation.

**Results**

RT-QuIC assays were performed on sporadic HPD (sHPD) samples comprising tissues diluted in PBS or RIPA buffer. Fluorescence intensities were measured in relative fluorescence units. RT-QuIC reaction samples with PBS buffer were used as a reference (Figs. S2a and S2b). We focused only on MM1-sCJD cases to exclude any *PRNP* gene codon 129 genotype or PrP^Sc^-type effects on the RT-QuIC seeding response. Figures S2a and S2b show that RT-QuIC assays of scalp samples were 100% positive at a dilution of 10^−4^ using PBS. The control group demonstrated no responses. RT-QuIC was performed on scalp samples from patients 10 and 13 using a RIPA buffer. There was no response to RT-QuIC using this buffer in the patient and control groups. The RT-QuIC response was inhibited by RIPA buffer.

**Supplementary Fig. S4 online.** **Prion seeding distribution activity across various scalp regions**


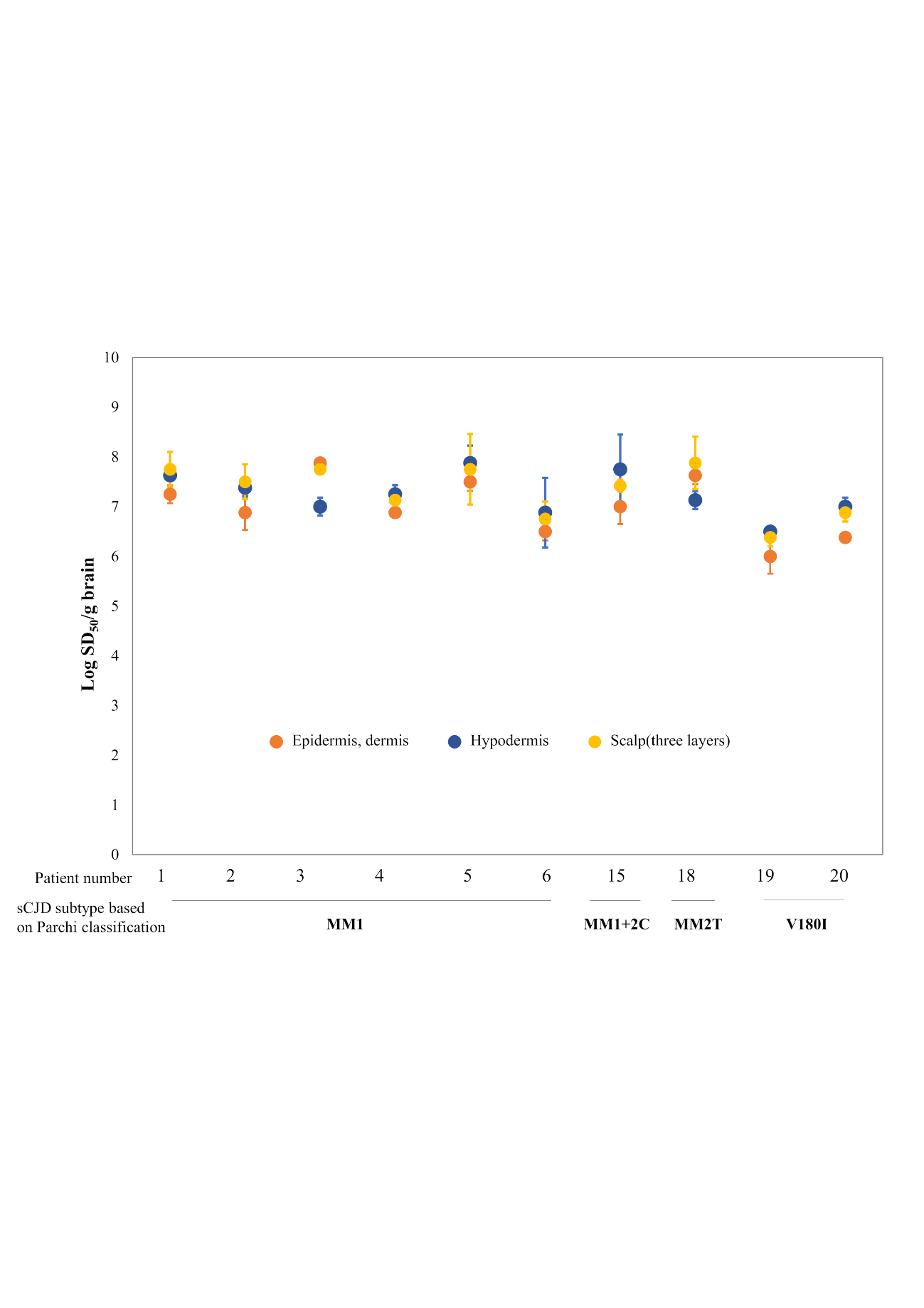
Quadruplicate RT-QuIC reaction samples were seeded with 10 µL of scalp tissue with serial 10^−3^–10^−8^ dilutions. Negative control reaction samples were seeded with a 10^−1^–10^−6^ dilution. The y-axis represents the logSD_50_/g tissues, and the x-axis represents the patient from which the sample was obtained.

**Method**

We measured LogSD_50_ in 10 scalp samples that contained all three scalp layers—the epidermis, dermis, hypodermis, and all layers—from 10 patients with HPD [MM1 (n = 6), MM2T (n = 1), MM1 + 2C (n = 1), and V180I (n = 2)]. Then, we analyzed the distributions of prion seeding activities in the epidermis, dermis, and hypodermis of scalp samples obtained from patients with HPD. A specialized knife cut the three-layer scalp samples into two samples: one containing the hair roots (epidermis and dermis) and the other containing the hypodermis (approximately 30–40 mg and 3 × 3 mm each). These samples were washed thrice in 1 × PBS and minced, then homogenized in 10% (weight/volume) PBS buffer for 2–4 min using a Power Masher 2 machine. The samples were centrifuged at 6,000 rpm for 2 min and stored at −80°C.

**Results**

Prion seeding activity was quantified across the epidermis, dermis, and hypodermis. RT-QuIC was conducted on 10 patients with HPD. The prion seeding activity was evenly distributed throughout the various skin layers. LogSD_50_ quantification showed almost equal prion seeding activity across the different skin areas, with minimal differences noted among the SD_50_ per gram of scalp across all three layers. The epidermis and dermis regions had the smallest values in the V180I cases (approximately 0.22). Prion seeding activity was larger in the MM1 cases (approximately 7.49) and largest in the MM1 + 2C (7.12) and V180I (6.44) cases. Prion seeding activity in the V180I cases had smaller values for epidermis and dermis than PSA in MM2T cases.

**Supplementary Fig. S5 online.** **RT-QuIC reactions at different temperatures**

**
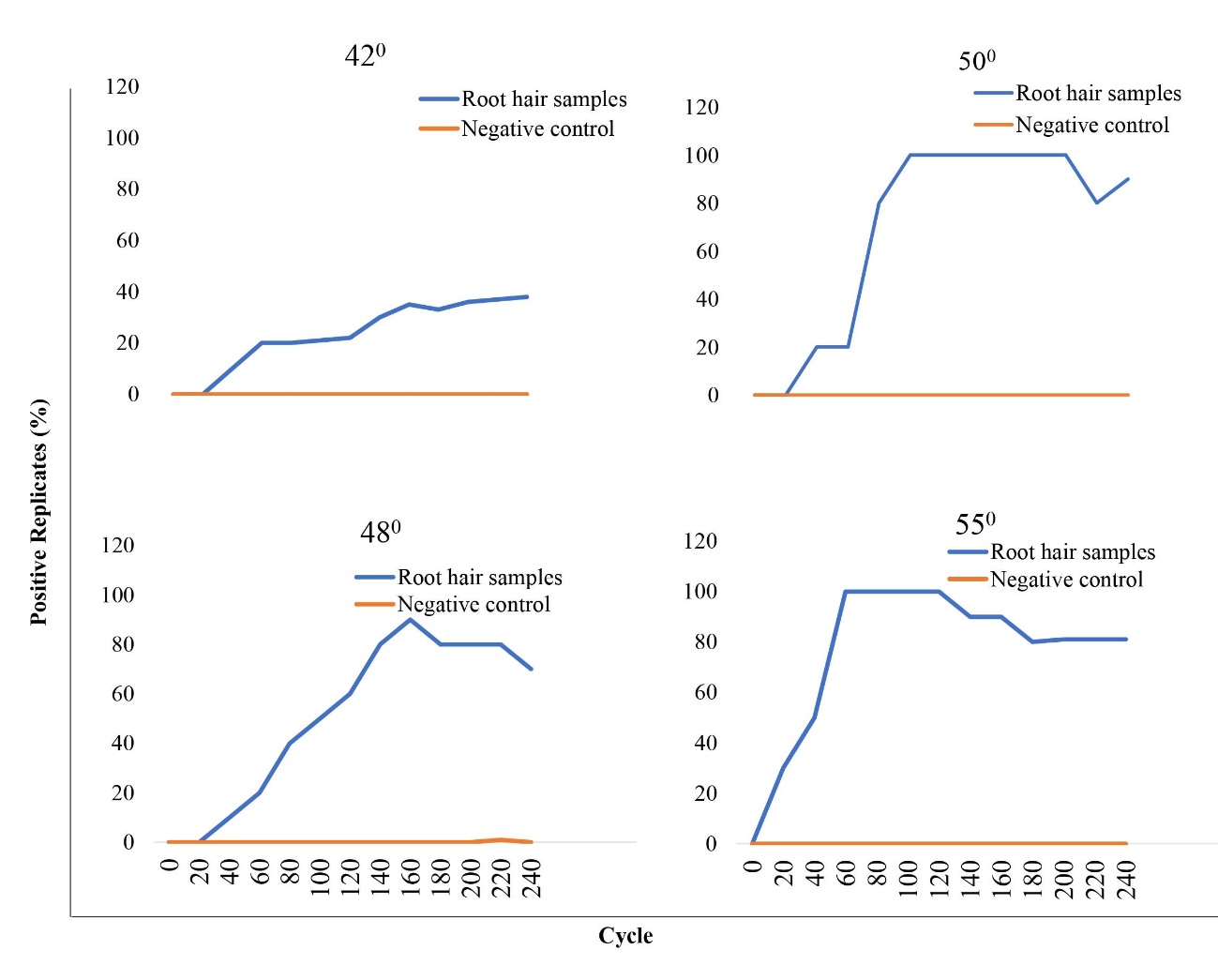
**

We used the RT-QuIC assay on hair root samples from patients with HPD at 42°C, 48°C, 50°C, and 55°C. The replicate reaction samples were incubated at the above-referenced temperatures. The increase in the average normalized ThT fluorescence of replicate wells (y-axis) is plotted as a function of the cycle (x-axis).

**Method**

Root hair samples were collected from two patients with HPD and two controls (one patient with Alzheimer’s disease and the other with Lewy body dementia). The root hairs were homogenated using PBS (15 hair roots/120–200 μl PBS) after being washed three times in 100–200 μl PBS and diced. Hair root homogenates were prepared by mashing the short hair pieces for 2–4 min with a Power Masher 2 machine in ice-cold PBS. The samples were centrifuged for 5 min at 6,000 rpm at 4°C and used to seed (10 µL) quadruplicate reaction samples.

**Results**

We systematically investigated the effects of increasing the RT-QuIC reaction temperature, building upon previous research, which found that using a HarPrPSen (90–231) substrate and a 55°C reaction temperature increased the speed and sensitivity with which RT-QuIC detected sHPD seeds in CSF. Root hair samples were homogenated with PBS (15 hair roots/120–200 μl PBS) and seeded with the substrate for quadruplicate reaction samples in the RT-QuIC assay. ThT fluorescence was assessed by increasing the temperature from 42°C to 60°C (Fig. S3).

None of the negative control reaction samples seeded with root hair samples were positive within 250 cycles (data not shown) at temperatures up to 60°C. A results comparison from four independent experiments (performed at 42°C, 48°C, 50°C, and 55°C) using hair root samples confirmed that, at higher temperatures, less time was needed to reach the maximum ThT fluorescence for each seed (Fig. S3).

At 50°C and 55°C, the fluorescence quickly reached its highest level. At 55°C, the sensitivity was maximized, and, in a short time, maximum ThT was reached. At 48°C, the RT-QuIC response took longer to reach maximum ThT fluorescence at cycle 160. At 42°C, the RT-QuIC reaction showed no fluorescence.

**Supplementary Table S1 online. Sensitivity and specificity of skin RT-QuIC assays in previous reports**

| **Year/Authors** | **Study type** | **Number of patients** | **Sensitivity and specificity** |
| --- | --- | --- | --- |
| 2017 Orrú CD, et al. (1) | Prospective | sporadic prion disease (n = 21), variant CJD (n = 2), and non-HPD (n = 15) | 100% and 100% specificity sCJD |
| 2020 Mammana A, et al. (2) | Prospective | punch biopsy skin samples (n = 71) from 35 patients with sporadic HPD | 89% sensitivity and 100% specificity |
| 2021 Honda H, et al. (3) | Prospective | Skin biopsy sample (n = 1) from one patient with sporadic HPD | 100% sensitivity and 100% specificity |
| 2021 Kang Xiao, et al. (4) | Prospective | sporadic HPD (n = 35), genetic HPD (n = 3), and non-HPD (n = 14) | 91.2% sensitivity and 86% specificity for sporadic HPD.  One gCJD case was positive |

HPD: human prion disease, CJD: Creutzfeldt–Jakob disease, sCJD: sporadic type of CJD,

gCJD: genetic type of CHD

1. Orrú CD, et al. Prion seeding activity and infectivity in skin samples from sporadic Creutzfeldt–Jakob disease patients. *Sci Transl Med* 2017; 9(417): eaam7785.
2. Mammana A, et al. Detection of prions in skin punch biopsies of Creutzfeldt–Jakob disease patients. *Ann Clin Transl Neurol* 2020; 7(4): 559.
3. Honda H, et al. Abnormal prion protein deposits with high seeding activities in the skeletal muscle, femoral nerve, and scalp of an autopsied case of sporadic Creutzfeldt–Jakob disease. *Neuropathology* 2021; 41(2): 152.
4. Xiao K, et al. Validation and application of skin RT-QuIC to patients in China with probable CJD. *Pathogens* 2021; 10(12): 1642.

**Supplementary Table S2 online. The sensitivity of cerebrospinal fluid (CSF) biomarkers and the hair root QuIC assay in human patients with prion disease**

| **Classification** | **n** | **CSF** | | | | **Hair root** | **MR-diffusion image** |
| --- | --- | --- | --- | --- | --- | --- | --- |
|  |  | **14-3-3 protein** | **Total tau protein** | **RT-QuIC**  **hu-1^st^-generation QuIC** | **RT-QuIC**  **2^nd^- generation QuIC** | **RT-QuIC**  **Revised-2^nd^**  **generation QuIC** |  |
| Sporadic HPD | 139 | 86.3% | 89.2% | 85.6% | 85.6% | 56.1% | 100% |
| Genetic HPD | 37 | 70.3% | 75.7% | 32.4% | 32.4% | 37.8% | 100% |
| Acquired HPD | 1 | 100% | 100% | 100% | 100% | 100% | 100% |

All patients had the codon 129 methionine homozygous (MM) genotype.

**Supplementary Table S3 online. The sensitivity of CSF biomarkers and the hair root QuIC assay in genetic prion disease**

| **Classification** | **n** | **CSF** | | | | **Hair root** | **MR-diffusion image (%)** |
| --- | --- | --- | --- | --- | --- | --- | --- |
|  |  | **14-3-3 protein (%)** | **Total tau protein (%)** | **RT-QuIC**  **(%)**  **hu-1^st^-generation QuIC** | **RT-QuIC**  **(%)**  **2^nd^- generation QuIC** | **RT-QuIC (%)**  **Revised 2^nd^-**  **generation**  **QuIC** |  |
| GSS (P102L) | 1 | 0 | 0 | 0 | 0 | 0 | 100 |
| Octapeptide repeat insertion | 1 | 0 | 0 | 0 | 0 | 0 | 100 |
| gCJD (V180I) | 22 | 68.20 | 77.30 | 4.50 | 4.50 | 13.60 | 100 |
| gCJD (E200K) | 6 | 100 | 100 | 100 | 100 | 100 | 100 |
| gCJD (M232R) | 6 | 83 | 83 | 83 | 83 | 83 | 100 |
| gCJD (V180I + M232R) | 1 | 100 | 100 | 0 | 0 | 0 | 100 |

n: number

Genetic HPD was divided into three subtypes: (Gerstmann–Sträussler–Scheinker Syndrome [GSS], genetic Creutzfeldt–Jakob disease [CJD], and octapeptide repeat insertion).

All patients had the codon 129 methionine homozygous (MM) genotype.

**Supplementary Table S4 online. Biomarker and the hair root QuIC assay sensitivity in definite cases of human prion disease**

| **Age at onset** | **Sex** | **CSF** | | | | **Hair root QuIC** | **Diagnosis** |
| --- | --- | --- | --- | --- | --- | --- | --- |
|  |  | **14-3-3 protein** | **Total tau protein** | **hu-1^st^-generation**  **QuIC assay** | **2^nd^-generation**  **QuIC assay** |  |  |
| early 80s | Male | Negative | Negative | Positive | Positive | Positive | sCJD |
| early 60s | Male | Positive | Positive | Positive | Positive | Positive | sCJD (MV2) |
| mid 80s | Male | Positive | Positive | Negative | Negative | Positive | gCJD (V180I) |
| mid 60s | Male | Positive | Positive | Positive | Positive | Positive | sCJD |
| early 70s | Male | Positive | Positive | Positive | Positive | Positive | sCJD |
| early 80s | Female | Negative | Negative | Positive | Positive | Positive | sCJD (MM2) |
| mid 70s | Female | Positive | Positive | Positive | Positive | Positive | gCJD (M232R) |
| mid 70s | Male | Positive | Positive | Positive | Positive | Positive | sCJD (MM1 + 2C) |
| mid 60s | Male | Positive | Positive | Positive | Positive | Positive | sCJD (MM1 + 2) |
| late 60s | Female | Negative | Negative | Positive | Positive | Positive | sCJD (MM1 + 2) |
| mid 70s | Male | Positive | Positive | Positive | Positive | Positive | sCJD (MM1 + 2) |
| early 70s | Female | Negative | Positive | Positive | Positive | Positive | sCJD (MM1) |

*All cases showed typical human prion disease findings on MRI diffusion-weighted images.

CJD: Creutzfeldt–Jakob disease, sCJD: sporadic type of CJD, gCJD: genetic type of CJD

**Supplementary Table S5 online. Endpoint RT-QuIC assay in native scalp layers obtained from human patients with prion disease**

| **Patient number** | **log SD_50_/g tissue of scalp (three layers) (mean ± SD)** | **log SD_50_/g tissue of scalp (epidermis and dermis) (mean ± SD)** | **log SD_50_/g tissue of scalp (hypodermis) (mean ± SD)** |
| --- | --- | --- | --- |
| 1 | 7.75 ± 0.35 | 7.25 ± 0.00 | 7.63 ± 0.18 |
| 2 | 7.5 ± 0.35 | 6.88 ± 0.18 | 7.38 ± 0.53 |
| 3 | 7.75 ± 0.00 | 7.88 ± 0.17 | 7.00 ± 0.00 |
| 4 | 7.13 ± 0.18 | 6.88 ± 0.18 | 7.25 ± 0.00 |
| 5 | 7.75 ± 0.71 | 7.50 ± 0.35 | 7.88 ± 0.18 |
| 6 | 6.75 ± 0.35 | 6.50 ± 0.70 | 6.88 ± 0.18 |
| 15 | 7.42 ± 0.12 | 7.00 ± 0.70 | 7.75 ± 0.35 |
| 18 | 7.88 ± 0.53 | 7.63 ± 0.18 | 7.13 ± 0.18 |
| 19 | 6.38 ± 0.18 | 6.00 ± 0.00 | 6.5 ± 0.35 |
| 20 | 6.88 ± 0.18 | 6.38 ± 0.18 | 7.00 ± 0.00 |

**Supplementary Table S6 online. The sensitivity of hair root RT-QuIC measures in patients with human prion disease (HPD) unable to undergo CSF testing**

| **Patient number** | **Age at onset** | **Sex** | **Hair root QuIC** |
| --- | --- | --- | --- |
| 1 | mid 80s | female | positive |
| 2 | mid 70s | female | positive |
| 3 | late 60s | male | positive |
| 4 | early 80s | female | positive |
| 5 | late 70s | female | positive |
| 6 | early 50s | male | positive |
| 7 | late 80s | female | positive |
| 8 | early 80s | female | negative |
| 9 | early 80s | female | negative |
| 10 | early 70s | male | negative |

All cases were identified using typical imaging technology. MRI diffusion-weighted images revealed high-signal areas in the cortex and basal ganglia.

Considering the test’s high significance, the following sHPD patients were subjected to further examination:

1) Patients with sHPD from 2021–2023 who provided written informed consent for participation.

2) Patients with severe lumbar spine deformities that precluded CSF testing, patients who refused CSF testing, and patients with severe myoclonus, for whom CSF testing would be too risky to perform.

3) Patients with more than 15 hair follicles and a large area of accessible scalp tissue after bathing, washing, or brushing their hair.

4) Patients who demonstrated the typical sHPD time course.

Consequently, the hair root RT-QuIC assay was performed in 10 patients with sHPD.
